# A Walk to Remember: Anesthetic Management of a Supercentenarian with Hip Fracture and Review of Literature

**DOI:** 10.7759/cureus.95216

**Published:** 2025-10-23

**Authors:** Kartik Sonawane, Payal Gursahani, Palanichamy Gurumoorthi, Jagannathan Balavenkatasubramanian

**Affiliations:** 1 Anesthesiology and Perioperative Medicine, Ganga Medical Centre and Hospitals Pvt. Ltd., Coimbatore, IND

**Keywords:** combined spinal-epidural, enhanced recovery, frailty, geriatric anesthesia, hip fracture, peng block, sifi block, supercentenarian

## Abstract

The steadily increasing global life expectancy has led to an unprecedented rise in the number of centenarians and supercentenarians (over 110 years) presenting for surgical procedures. While this demographic shift is a triumph of modern medicine, it also poses unique perioperative challenges. Such populations represent an extreme physiological spectrum, characterized by limited organ reserve, altered pharmacokinetics, and unpredictable responses to anesthetic agents. Yet, they often present with urgent surgical conditions such as hip fractures, where timely intervention is critical to preserve function and independence. The anesthetic management of such patients requires a meticulous, physiology-driven strategy that balances efficacy with safety while facilitating early mobilization to maintain independence. We report the anesthetic management of a 111-year-old man, the oldest trauma surgery patient at our institution, who underwent closed reduction and internal fixation of an intertrochanteric fracture.

This patient had no major comorbidities but carried the risk of extreme age-related frailty. We employed a combined spinal-epidural anesthesia (CSEA) technique with an ultra-low-dose intrathecal injection (0.6 mL of 0.5% heavy bupivacaine), customized according to our institutional age-dose protocol. A preoperative pericapsular nerve group (PENG) block facilitated pain-free positioning for the neuraxial anesthesia. Hemodynamic stability was proactively maintained with a prophylactic low-dose norepinephrine infusion, guided by a “mean arterial pressure ≈ patient’s age” principle. Postoperative recovery was supported with a supra-inguinal fascia iliaca (SIFI) block as a part of a multimodal, opioid-sparing strategy, embedded within our enhanced recovery after surgery (ERAS) protocol. This approach enabled mobilization by postoperative day 3 and discharge on day 7, a benchmark for functional recovery in a supercentenarian. No delirium or complications were observed at the 30-day follow-up.

This case, a true “Walk to Remember,” is contextualized by a literature review highlighting neuraxial anesthesia (low-dose spinal anesthesia, CSEA, and continuous spinal anesthesia {CSA}), peripheral blocks (PENG and SIFI), and proactive hemodynamic optimization in centenarians and supercentenarians. This is among the few documented reports of anesthesia in a supercentenarian worldwide. It reinforces that chronological age alone should not preclude surgery when careful physiological assessment, evidence-based anesthetic techniques, and multidisciplinary perioperative care guide management.

## Introduction

The global population is undergoing a profound demographic shift, with centenarians, particularly supercentenarians (aged 110 years or older), emerging as the fastest-growing age group [1]. United Nations projections estimate that by 2050, over 3.7 million individuals will be aged 100 years or older [1]. This longevity is driven by advances in nutrition, vaccination, the control of infectious and chronic diseases, and innovations in surgical and intensive care [2,3]. However, increased life expectancy is accompanied by a rise in age-related conditions such as osteoporosis, frailty, and fragility fractures, posing significant challenges for anesthesiologists in perioperative settings [4,5].

Hip fractures are among the most severe injuries in older adults, associated with the loss of independence, extended hospitalization, and elevated mortality [6,7]. The one-year mortality rate following hip fracture surgery in octogenarians and nonagenarians ranges from 20% to 30%, with even higher risks in centenarians [6,8-10]. Timely surgical fixation, multimodal analgesia (MMA), and early mobilization are crucial for optimizing outcomes; however, anesthetic management is pivotal in determining perioperative success in this vulnerable population [11,12].

Supercentenarians present unique anesthetic challenges due to age-related physiological changes that alter pharmacokinetics and pharmacodynamics [13,14]. Reduced cardiovascular reserve, driven by arterial stiffness and impaired baroreflex sensitivity, limits tolerance to hemodynamic shifts [15,16]. Respiratory function declines with decreased chest wall compliance and vital capacity, increasing risks during sedation or general anesthesia (GA) [17,18]. Impaired renal and hepatic clearance prolongs drug half-lives, heightening toxicity risks [13,19]. The central nervous system’s increased sensitivity to sedatives and local anesthetics necessitates precise dosing to avoid complications [14,20]. These factors demand an ultracautious, physiology-driven, and titratable anesthetic strategy.

Over the past two decades, regional anesthesia (RA) techniques, such as low-dose spinal anesthesia, combined spinal-epidural anesthesia (CSEA), and continuous spinal anesthesia (CSA), have gained prominence for hip fracture surgery in older adults due to their ability to minimize hemodynamic instability [21,22]. When paired with peripheral nerve blocks, such as the fascia iliaca or pericapsular nerve group (PENG) block, these techniques enhance analgesia, reduce opioid use, preserve cognitive function, and support enhanced recovery pathways [23-25]. Although centenarian- and supercentenarian-specific literature is limited to case reports and small series, these consistently affirm the safety and efficacy of titrated, protocol-driven RA [26,27].

We report the successful anesthetic management of a 111-year-old man, the oldest trauma surgery patient at our institution, who underwent closed reduction and internal fixation of an intertrochanteric femur fracture. This case highlights core principles of geriatric anesthesia: individualized dosing, proactive hemodynamic support, opioid-sparing MMA, and adherence to enhanced recovery after surgery (ERAS) protocols. Ultimately, it reflects not just the science of anesthesia but the art of giving a supercentenarian the dignity of mobility, a walk to remember. We also contextualize this experience within a review of the literature to provide educational value and practical insights for clinicians faced with similar scenarios.

## Case presentation

Patient presentation

**Figure 1:**
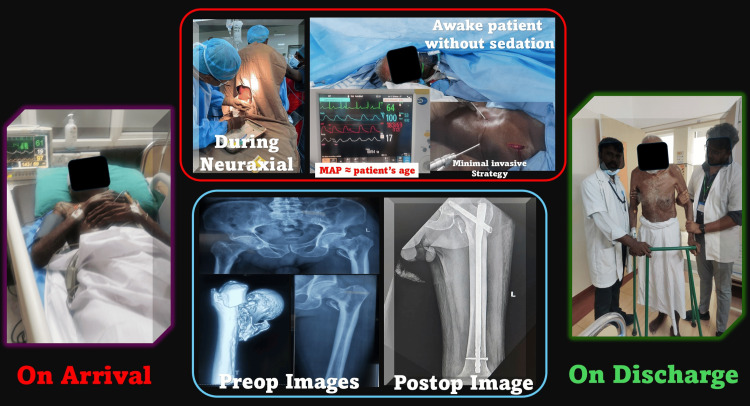
Perioperative course of a 111-year-old centenarian undergoing hip fracture fixation under regional anesthesia. The sequence of clinical images depicts the patient’s journey from admission to discharge. On arrival, the patient was monitored and stabilized before surgery. Intraoperative images show the administration of combined spinal-epidural anesthesia with stable hemodynamics, and the patient remained awake without sedation, maintained with a minimal invasive strategy. Preoperative radiographs confirmed an intertrochanteric femur fracture, and postoperative radiographs demonstrated successful fixation with intramedullary nailing. The final image shows the patient mobilized with support at discharge on postoperative day 7, highlighting functional recovery. MAP, mean arterial pressure; Preop, preoperative; Postop, postoperative Source: this figure was created by the first author, KS.

A 111-year-old man presented to our institution’s emergency department following a low-energy fall at home, resulting in an intertrochanteric femur fracture confirmed by X-ray (Figure 1). His medical history included hypertension (controlled with amlodipine 5 mg daily), mild cognitive impairment, and no documented cardiac or pulmonary disease. Preoperative assessment revealed a frail phenotype (Fried frailty score: 3/5) but preserved organ function: blood pressure, 143/69 mmHg; heart rate, 61 beats per minute (bpm); oxygen saturation, 97% on room air; and normal renal (creatinine: 1.0 mg/dL) and hepatic function (normal liver enzymes). Lumbar spine imaging confirmed the preservation of intervertebral spaces, facilitating the option of neuraxial anesthesia. His American Society of Anesthesiologists (ASA) classification was III, and he expressed a strong desire to regain mobility to maintain independence.

Given the patient’s age and frailty, a multidisciplinary team comprising an anesthesiologist, orthopedician, geriatrician, and critical care specialist convened to formulate a perioperative strategy. A consensus favored a physiology-driven approach incorporating RA as an adjunct to MMA, titrated neuraxial dosing according to our institutional age-dose protocol (Table 1), supported by a perioperative care pathway that emphasizes proactive hemodynamic monitoring and early mobilization within an ERAS framework.

**Table 1:** Age-adjusted institutional spinal anesthesia dosing protocol for hip fracture surgery. CSF, cerebrospinal fluid; CSEA, combined spinal-epidural anesthesia; CSA, continuous spinal anesthesia; LA, local anesthetic; ERAS, enhanced recovery after surgery

Age Group	Spinal Anesthesia Volume (0.5% Heavy Bupivacaine)	Adjuvants (Optional)	Key Notes
<60 years	2.5-3.0 mL	Fentanyl 20-25 µg or morphine 100-150 µg	Standard dosing, adequate CSF volume, healthy reserve, higher tolerance, and ERAS considerations apply
60-70 years	2.2-2.5 mL	Fentanyl 15-20 µg	↓ CSF volume begins with moderate sensitivity, and the dose should be adjusted downward for smaller stature
70-80 years	1.8-2.2 mL	Fentanyl 10-15 µg	More pronounced CSF volume decline, physiological changes, ↑ cephalad spread risk, and increased hemodynamic variability
80-90 years	1.2-1.8 mL	Fentanyl ≤10 µg	Very high sensitivity, titrate carefully, and consider CSEA if surgery may be prolonged
90-100 years	0.8-1.2 mL	Fentanyl ≤10 µg	Maximum caution, ↓ CSF volume, ↓ cardiovascular reserve, safest with CSEA/CSA for titration, and exaggerated sensitivity to LAs
100-110 years	0.6-0.8 mL	Fentanyl ≤10 µg	Ultra-low dosing is essential, and CSEA/CSA for supplementation is recommended
>110 years	0.4-0.6 mL	Fentanyl ≤10 µg	Supercentenarians require extreme titration, with proactive vasopressor infusion mandatory

Preoperative analgesia

**Figure 2:**
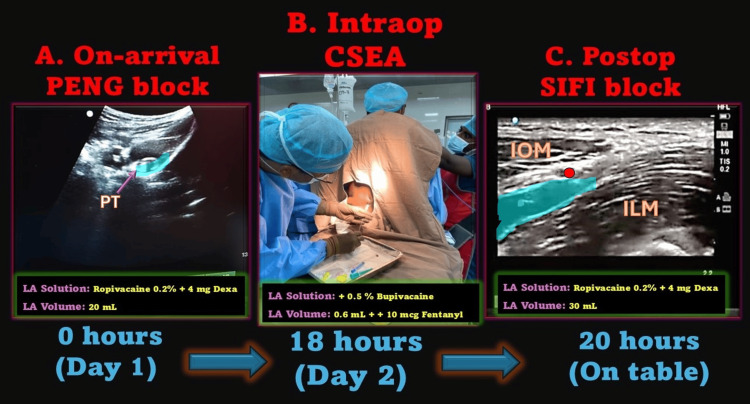
Perioperative regional anesthesia strategy in a 111-year-old supercentenarian. (A) On arrival, an ultrasound-guided pericapsular nerve group (PENG) block was performed using 20 mL of 0.2% ropivacaine with 4 mg dexamethasone, providing effective preoperative analgesia. The blue area shows local anesthetic spread under the psoas tendon (PT). (B) Intraoperatively, combined spinal-epidural anesthesia (CSEA) was administered with 0.6 mL of 0.5% hyperbaric bupivacaine and 10 µg fentanyl, achieving adequate surgical anesthesia while maintaining hemodynamic stability. (C) At the conclusion of surgery, a postoperative supra-inguinal fascia iliaca (SIFI) block was performed using 30 mL of 0.2% ropivacaine with 4 mg dexamethasone to ensure prolonged analgesia and facilitate early mobilization. The blue area denotes local anesthetic spread between the internal oblique (IOM) and iliacus muscle (ILM), under the fascia iliaca, located below the deep circumflex iliac artery (shown as a red circle). LA, local anesthetic; Dexa, dexamethasone; Intraop, intraoperative; Postop, postoperative Source: this figure was created by the first author, KS.

Upon arrival, an ultrasound-guided PENG block was performed using 20 mL of 0.2% ropivacaine and 4 mg of dexamethasone (Figure 2A). This block provided immediate and sustained analgesia, obviating systemic opioid use and ensuring pain-free rest overnight. Importantly, it eliminated the need for a “positioning block” before neuraxial anesthesia on the next day, a major advantage in elderly patients with hip fracture, where pain often hinders safe positioning.

Intraoperative management

Inside the operating room, standard ASA monitoring (electrocardiogram, pulse oximetry, and capnography) was instituted. Invasive arterial blood pressure monitoring was established for beat-to-beat assessment, considering the patient’s extreme age and limited physiological reserve. Ultrasound examination revealed markedly sclerotic arterial walls, a reflection of vascular ageing, yet cannulation was achieved without difficulty. Considering the patient’s extreme age and the nature of surgery, additional measures included the use of a body warmer and fluid warmer to prevent perioperative hypothermia.

CSEA was administered in the sitting position at the L3-L4 interspace, using 0.6 mL of 0.5% hyperbaric bupivacaine and 10 µg of fentanyl intrathecally, followed by epidural catheter placement (Figures 1, 2B). A block height of T12 was achieved, and 30 minutes later, an epidural top-up of 6 mL 0.5% bupivacaine extended surgical anesthesia seamlessly. Hemodynamic stability was proactively maintained with a continuous low-dose norepinephrine infusion (1 mL/hour), titrated to maintain mean arterial pressure (MAP) close to baseline (“MAP ≈ patient’s age” principle) (Figure 1). Invasive arterial monitoring ensured real-time hemodynamic assessment, and forced-air warming maintained normothermia (36.5°C-37°C). Total blood loss was approximately 250 mL, managed with one unit of leukodepleted packed red blood cells and 1 L of crystalloids. Oxygen was delivered at 4 L/minute via a Hudson mask, ensuring adequate oxygenation without the need for intraoperative sedation. Urine output remained satisfactory throughout, reflecting stable renal perfusion.

Postoperative course

At the conclusion of surgery, the epidural catheter was removed, and a supra-inguinal fascia iliaca (SIFI) block was performed using 30 mL of 0.2% ropivacaine with 4 mg dexamethasone to provide extended postoperative analgesia (Figure 2C). The patient was monitored in the high-dependency unit for 24 hours before transfer to the ward. Pain control was excellent without the use of systemic opioids, preserving cognitive clarity and respiratory function. Early physiotherapy was initiated per our institutional ERAS protocol (Table 2). The patient was mobilized with assistance on postoperative day 3. He was discharged home in stable condition on postoperative day 7, marking a successful recovery in one of the oldest trauma patients reported to date. No delirium or complications were noted at 30-day follow-up. Written informed consent was obtained from the patient’s legally authorized representative for the publication of this case report and any accompanying images. Patient anonymity has been preserved, and no identifying information has been disclosed.

**Table 2:** Institutional protocol for the perioperative management of ultra-elderly patients with hip fracture. USG, ultrasound-guided; PENG, pericapsular nerve group; HDU, high-dependency unit; SIFI, supra-inguinal fascia iliaca; POD, postoperative day; ERAS, enhanced recovery after surgery

Step	Protocol Element	Details/Our Practice
1. On-arrival analgesia	Peripheral nerve block	USG-guided PENG block with 20 mL 0.25% ropivacaine → pain relief, positioning, and ↓ opioid use
2. Preoperative preparation	Focused optimization	Minimal but targeted laboratory tests, hydration correction, and HDU monitoring; avoid unnecessary delays
3. Choice of neuraxial anesthesia	Combined spinal-epidural (preferred)	Intrathecal doses of 0.6-1.0 mL of 0.5% heavy bupivacaine (age-stratified); epidural catheter for titration
	Continuous spinal (alternative)	Microcatheter with fractionated dosing (0.2-0.4 mL increments) and precise titration in frail/high-risk patients
	Epidural alone (selective)	If spinal is contraindicated, slow titration, mainly for analgesic role
4. Hemodynamic management	Vasopressor strategy	Prophylactic norepinephrine infusion (1-2 mL/hour; 8 mg/50 mL dilution) started at spinal injection; avoid fluid overload
5. Intraoperative analgesia	Epidural supplementation	Incremental 3-5 mL 0.25% bupivacaine/lignocaine as required
6. Postoperative analgesia	Regional blocks	SIFI block with 25-30 mL 0.25% ropivacaine; no opioids used
7. ERAS principles	Early recovery strategy	Active warming, multimodal non-opioid analgesia, physiotherapy from POD 2-3, and ambulation by POD 3
8. Discharge plan	Enhanced recovery	Target discharge at POD 5-7; follow-up for functional independence

## Discussion

The anesthetic management of supercentenarians requires a nuanced balance of clinical precision and individualized care. While chronological age is a marker, frailty, physiological reserve, and comorbidities are the primary drivers of perioperative risk [4,5]. Our 111-year-old patient’s preserved organ function enabled an RA-based approach, but the extreme age necessitated a conservative, physiology-driven strategy guided by institutional protocols [2]. Age-related physiological changes, such as altered pharmacokinetics and heightened neural sensitivity, amplify the challenges of anesthesia in supercentenarians (Table 3).

**Table 3:** Age-related physiological changes, their impact on neuraxial anesthesia, and clinical implications in elderly and super-elderly patients. CSF, cerebrospinal fluid; MAP, mean arterial pressure; CSEA, combined spinal-epidural anesthesia; CSA, continuous spinal anesthesia; GA, general anesthesia; RA, regional anesthesia; PENG, pericapsular nerve group

System	Age-Related Changes	Impact on Neuraxial Anesthesia	Clinical Implications/Modifications
Central nervous system	↓ Neuronal reserve, ↑ sensitivity to local anesthetics, ↓ CSF volume, and altered CSF dynamics	Greater cephalad spread of intrathecal drugs and ↑ risk of high spinal	Use lower intrathecal doses (0.6-1.0 mL of heavy bupivacaine), favoring titratable techniques (CSEA and CSA)
Spinal anatomy	Spinal stenosis, calcification, scoliosis, and ↓ intervertebral space	Technical difficulty with needle placement and unpredictable spread	Use ultrasound guidance when needed and consider CSEA/CSA to titrate
Cardiovascular	Arterial stiffness, ↓ baroreceptor sensitivity, diastolic dysfunction, and ↓ β-receptor responsiveness	Severe hypotension after sympathectomy and poor compensation	Initiate prophylactic norepinephrine infusion, restrict fluids, and maintain MAP near baseline
Respiratory	↓ Elastic recoil, ↓ vital capacity, ↑ closing volume, and ↑ risk of atelectasis	Reduced reserve for sedation or GA and prone to hypoxemia if oversedated	Prefer RA over GA, avoid deep sedation, and use opioid-sparing analgesia
Renal	↓ GFR, ↓ renal perfusion, and altered drug clearance	Prolonged action of anesthetics and opioids	Avoid nephrotoxic drugs, titrate doses, and maintain renal perfusion with stable MAP
Hepatic	↓ Hepatic mass and blood flow and ↓ phase I metabolism	Reduced clearance of amide local anesthetics and sedatives	Reduce cumulative dosing and avoid long-acting systemic sedatives
Musculoskeletal	Osteoporosis and fragile positioning	Pain during neuraxial positioning	Use pre-procedural nerve blocks (e.g., PENG) to aid positioning
Cognitive	Baseline cognitive decline and vulnerability to delirium	Risk of postoperative cognitive dysfunction (POCD) and delirium	Avoid benzodiazepines, minimize opioids, and prefer RA + multimodal non-opioid analgesia
Thermoregulatory	↓ Shivering threshold and ↓ temperature control	Increased hypothermia risk intraoperatively	Use active warming to maintain normothermia and prevent complications

Review of literature

To contextualize our approach, it is important to review the available evidence on anesthesia in the very elderly, including octogenarians, nonagenarians, centenarians, and supercentenarians. The perioperative management of elderly patients undergoing hip fracture surgery has been extensively studied in octogenarians, less so in nonagenarians, and only rarely in centenarians and supercentenarians [10,28]. The literature highlights a progressive shift in anesthetic techniques with advancing age, emphasizing dose reduction, multimodal analgesia (MMA), and proactive hemodynamic support as critical strategies for optimizing outcomes [2,29].

Octogenarians (80-89 Years)

This age group dominates the geriatric anesthesia literature, with large cohort studies and randomized trials showing that neuraxial anesthesia, particularly spinal anesthesia or CSEA, reduces postoperative pulmonary complications, thromboembolism, and mortality compared to GA [7,8,21,30-35]. Intrathecal doses of 1.5-2.0 mL of 0.5% hyperbaric bupivacaine are typically sufficient, providing effective anesthesia while minimizing hemodynamic instability [32]. ERAS protocols, including early mobilization and opioid-sparing analgesia, significantly reduce hospital length of stay and mortality in this cohort [12,36-38]. However, prevalent comorbidities such as heart failure and chronic obstructive pulmonary disease necessitate rigorous preoperative optimization and intraoperative monitoring [10,29]. Moreover, RA improves pain control, reducing opioid requirements compared to GA, thereby decreasing delirium and other opioid-related complications [39].

Nonagenarians (90-99 Years)

Studies in this age group are less abundant, primarily consisting of retrospective series and case reports that confirm the feasibility of RA [5,40]. Ultra-low intrathecal doses (1.0-1.5 mL of 0.5% bupivacaine) and CSA are effective, allowing for the precise titration of block height to minimize hemodynamic fluctuations [22,41-43]. The heightened risk of postoperative delirium in nonagenarians underscores the value of opioid-sparing MMA and early mobilization to preserve cognitive function [9,44-46].

Centenarians (100-109 Years)

Evidence is limited to case reports and small series yet consistently supports RA’s safety [6,26-28,47-51]. A 100-year-old COVID-19-positive patient underwent hip hemiarthroplasty with 2.5 mL of 0.5% preservative-free bupivacaine, maintaining stable hemodynamics without complications [49]. Another had hip fracture surgery with 8 mg (1.6 mL) of 0.5% hyperbaric bupivacaine, achieving stable outcomes and discharge on day 7 [50]. A third 100-year-old received spinal anesthesia with a virtual reality adjunct for hip arthroplasty, avoiding delirium [51]. Combined neuraxial and peripheral nerve block strategies are frequently advocated to reduce systemic drug exposure and enhance recovery [40,52].

Supercentenarians (≥110 Years)

Reports are exceedingly rare but demonstrate RA’s feasibility [53-55]. To date, there are no large series. A supercentenarian received unilateral spinal anesthesia with norepinephrine support for surgery, demonstrating feasibility at extreme age [54]. Another 111-year-old woman underwent hip arthroplasty with low-dose spinal anesthesia [55]. These cases suggest that, with careful protocolization, surgery remains viable even at extreme ages [31,53]. Our case of a 111-year-old trauma patient thus represents one of the very few documented experiences of anesthesia in this demographic.

Summary

Table 4 summarizes key reports across these age groups, demonstrating the consistent safety and efficacy of RA in the very elderly. Across the eighth to eleventh decades, the literature consistently supports the following: (1) RA’s superiority over GA in reducing pulmonary complications, delirium, and recovery time [7,8,21]; (2) the progressive dose reduction of intrathecal bupivacaine, from ~2 mL in octogenarians to <1 mL in centenarians [32,43]; (3) CSA and CSEA’s role in providing hemodynamic stability through titration [22,41]; and (4) peripheral nerve blocks and ERAS protocols’ efficacy in minimizing opioids and enhancing recovery [36,40,45].

**Table 4:** Summary of reported literature on anesthesia strategies in elderly surgical patients (octogenarians to supercentenarians). RA, regional anesthesia; SA, spinal anesthesia; GA, general anesthesia; CSA, continuous spinal anesthesia; ERAS, enhanced recovery after surgery; VR, virtual reality; RCT, randomized controlled trial; LA, local anesthetic

Age Group	Study/Author (Year)	Anesthesia Type(s) Compared	Key Findings
Octogenarians (80-89)	Koval et al. (1999) [31]	Spinal versus GA	No difference in one-year mortality (~25%) or morbidity, as noted in an older study
	Errando et al. (2014) [32]	Low-dose spinal	Ultra-low dose (1.5-2 mL bupivacaine) is effective with minimal hypotension; safe for frail elderly
	Van Waesberghe et al. (2017) [21]	Neuraxial anesthesia (NA) versus GA	NA is associated with lower thromboembolism and shorter hospital stays, as shown in a meta-analysis of over 20 studies
	Brox et al. (2016) [33]	NA versus GA	Similar mortality; no specific recommendation for type
	Kowark et al. (2019) [30]	Spinal versus GA	NA reduced postoperative complications (e.g., pneumonia) by 20% in a cohort of >10,000 patients
	Neuman et al. (2021) [8]	Spinal versus GA	No superiority of spinal over GA for survival or delirium, similar 60-day mortality (~8%), and cognitive outcomes
	Cheung et al. (2023) [44]	Neuraxial versus GA	NA reduced pulmonary complications and mortality (OR: 0.75); improved recovery in a large meta-analysis of RCTs
	Chowdary et al. (2023) [34]	Spinal versus GA	NA linked to fewer postoperative complications (e.g., infections), retrospective cohort
	Sameer et al. (2023) [12]	Multimodal RA + ERAS	ERAS with RA reduced the length of stay by two days and mortality by 15%, in a prospective cohort
	Noji et al. (2024) [35]	Spinal versus GA	No difference in psoas muscle loss postoperatively; both are safe
	Li et al. (2025) [39]	GA versus RA	RA improved pain control and reduced opioids in a cohort study
Nonagenarians (90-99)	Döhler et al. (1999) [42]	CSA in high-comorbidity elderly	Safe in 154 cases is >80, low complication rate with titration
	Minville et al. (2006) [22]	Low-dose spinal versus CSA	CSA superior for titration, reduced hypotension, and RCT in the elderly is >80
	Rabinowitz et al. (2007) [41]	Paramedian versus midline approaches of CSA	Paramedian CSA is associated with an increased success rate
	Minville et al. (2008) [43]	Three techniques (including NA/GA)	NA minimized hypotension versus GA and elderly femoral neck fractures
	Acharya et al. (2022) [40]	Peripheral blocks + NA	Effective in a nonagenarian with coagulopathy; case report
	Wang et al. (2022) [47]	Surgical fixation outcomes	RA is preferred for reduced mortality in nonagenarians
	Fan et al. (2024) [9]	GA versus spinal	No difference in delirium incidence, meta-analysis
Centenarians (100-109)	Imbelloni et al. (2014) [26]	NA for hip surgery	Successful in a 107-year-old; low-dose spinal safe
	Cevik (2016) [50]	Spinal with 8 mg LA	The 90-minute surgery without hemodynamic incompetence resulted in stable outcomes
	Dick et al. (2017) [28]	Various (mostly RA)	RA safe; improved care in the database era but high mortality (~40%)
	Beathe and Memtsoudis (2020) [49]	Spinal with 2.5 mL LA	COVID-19-positive with stable hemodynamics with no postoperative complications
	Irianto et al. (2021) [48]	Regional (epidural) for hemiarthroplasty	Successful in a 100-year-old with fractures and stable outcomes
	Ledford et al. (2021) [51]	Adjunct VR + NA	Safe in centenarian hip arthroplasty and reduced sedation needs
	Abelleyra et al. (2023) [27]	Various (RA preferred)	RA associated with better functional recovery; systematic review and meta-analysis
	Jang et al. (2023) [10]	Cohort study	RA reduced mortality risk factors in multicenter data
Supercentenarians (≥110)	Tosun et al. (2015) [54]	Unilateral spinal	Successful in supercentenarian, ultra-low dose
	Sharma et al. (2020) [53]	Small gut obstruction for emergency laparoscopy	Short-acting anesthetic drugs in titrated quantities, better and faster recovery
	Wu et al. (2020) [55]	RA for arthroplasty	Walked out postoperatively at 111; emphasizes RA feasibility

Building upon these prior findings, our case demonstrates how these principles can be safely adapted to the supercentenarian population through protocolized, physiology-based perioperative management.

RA versus GA in supercentenarians

Neuraxial anesthesia, particularly CSEA, is consistently reported as the preferred approach for patients over 90 years of age due to its favorable safety profile [5,8]. Compared to GA, RA reduces postoperative delirium, pulmonary complications, and recovery time in centenarians and supercentenarians [21,9]. Studies highlight GA’s association with increased postoperative cognitive dysfunction and prolonged ventilation in the ultra-elderly [30,56]. RA, by contrast, minimizes systemic drug exposure, preserves respiratory function, and facilitates early mobilization [13,21]. Our use of CSEA provided rapid spinal onset and epidural flexibility, accommodating variable surgical durations while maintaining hemodynamic stability [22,42]. Combined with peripheral nerve blocks, this approach underscores RA’s superiority for hip fracture surgery in supercentenarians [40,57].

Ultra-low-dose intrathecal anesthesia: A cornerstone of safety

Neuraxial dosing in supercentenarians requires extreme caution due to reduced cerebrospinal fluid (CSF) volume and increased neural sensitivity, which narrows the therapeutic window [13,2]. Standard doses of 2.5-3.0 mL of 0.5% hyperbaric bupivacaine, used in younger adults, risk profound hypotension or high spinal block [32]. Our decision to use ultra-low intrathecal dosing was consistent with our institutional neuraxial protocol (Table 5), which was specifically designed for the ultra-elderly. Our protocol recommends 0.6 mL for patients over 110 years, as outlined in Table 1. Such age-related progressive dose reduction mitigates risks of cardiovascular collapse [43]. This “less is more” approach prevents catastrophic complications such as profound hypotension or high spinal block. The epidural component of CSEA allowed for intraoperative titration, thereby enhancing safety in this population [22].

**Table 5:** Institutional neuraxial anesthesia dosing protocol for geriatric patients. CSF, cerebrospinal fluid; ASA, American Society of Anesthesiologists

Technique	Drug and Dose	Rationale	Clinical Notes
Single-shot spinal anesthesia	0.6-1.0 mL of 0.5% heavy bupivacaine (with/without opioid adjuvant such as fentanyl 10-15 µg)	Reduced CSF volume in elderly → ↑ cephalad spread; avoids high spinal	Use the lower end (0.6-0.8 mL) in centenarians/supercentenarians/frail ASA IV-V; the higher end (~1.0 mL) for robust elderly
Combined spinal-epidural anesthesia (CSEA)	Intrathecal: 0.6-0.8 mL of 0.5% heavy bupivacaine + epidural catheter for titration with 3-5 mL 0.25% bupivacaine/lignocaine as needed	Provides rapid onset with a safety net of epidural supplementation	Preferred in centenarians; allows tailored duration and hemodynamic stability
Continuous spinal anesthesia (CSA)	Initial 0.2-0.4 mL of 0.5% isobaric/low-dose heavy bupivacaine; repeat 0.2-0.3 mL increments every 15-20 minutes via microcatheter	Fine titration → hemodynamic stability and prolonged anesthesia	Useful for frail/high-risk elderly and prolonged or unpredictable surgery
Epidural alone	3-5 mL aliquots of 0.25% bupivacaine/lidocaine every 10-15 minutes until the desired level	Slow onset and titratable block	Reserved when spinal is contraindicated (e.g., anticoagulation and severe spinal deformity)
Adjuvants (optional)	Fentanyl 10-15 µg intrathecal or dexmedetomidine 5-10 µg epidural	Enhances block quality and prolongs analgesia	Use cautiously; avoid systemic opioids; minimize sedation

Perioperative monitoring and thermal management

Meticulous monitoring is critical in supercentenarian anesthesia [29]. Invasive arterial pressure monitoring enables real-time hemodynamic assessment and proactive vasopressor titration, reducing risks of myocardial ischemia and renal dysfunction, making invasive monitoring a cornerstone of safety rather than a luxury [58,59]. Hypothermia, prevalent due to impaired thermoregulation, exacerbates coagulopathy and delays drug metabolism [60,61]. Forced-air warmers and fluid warmers, used in our case, align with best practices for maintaining normothermia and supporting hemodynamic stability [62,63].

Hemodynamic management: Proactivity over reactivity

Hypotension is the most feared complication of neuraxial anesthesia in centenarians/supercentenarians, potentially causing myocardial ischemia or cerebral hypoperfusion [43,59]. Proactive low-dose norepinephrine infusions, as used in our case, stabilize MAP and reduce delirium risk compared to reactive bolus therapy [64,65]. This approach reflects a shift toward preemptive hemodynamic management in frail patients [66,67]. Low-dose norepinephrine infusion stabilizes mean arterial pressure, preserves cerebral oxygenation, and reduces the incidence of delirium [64,65]. In our case, initiating norepinephrine at induction resulted in a stable intraoperative course with minimal fluctuations. This aligns with the emerging consensus that hemodynamic management in frail elderly patients should shift from “chasing drops in pressure” to “preempting instability.”

Role of peripheral nerve blocks in a multimodal strategy

Peripheral nerve blocks are increasingly recognized as essential adjuncts in the perioperative care of elderly patients with hip fractures [23,24]. By targeting the articular branches of the hip capsule, the PENG block provides profound analgesia with minimal motor involvement, making it superior to traditional femoral or fascia iliaca blocks for positioning and comfort [25]. In our case, the PENG block facilitated pain-free positioning for neuraxial placement and avoided the need for systemic opioids. The SIFI block extended postoperative pain control in our case, reducing opioid use and preserving cognitive function [57]. Meta-analyses confirm that multimodal RA reduces pulmonary complications, thromboembolism, and delirium, all of which are disproportionately fatal in older adults [45,46]. A comparative perspective of GA, RA, and hybrid techniques is summarized in Table 6, underscoring the superiority of regional strategies for pulmonary, cognitive, and recovery outcomes.

**Table 6:** Comparative perioperative impact of general anesthesia (GA), neuraxial anesthesia, and hybrid GA + regional techniques in elderly patients. RA, regional anesthesia; CSEA, combined spinal-epidural anesthesia; CSA, continuous spinal anesthesia; PNBs, peripheral nerve blocks; PENG, pericapsular nerve group; SIFI, supra-inguinal fascia iliaca; ERAS, enhanced recovery after surgery; POCD, postoperative cognitive dysfunction; PDPH, post-dural puncture headache

Parameter	General Anesthesia (GA)	Neuraxial Anesthesia (NA)	GA + Regional Blocks (Hybrid)	Clinical Take-Home
Airway and respiratory	Requires airway instrumentation; ↑ risk of aspiration, pneumonia, and atelectasis	Preserves spontaneous breathing; avoids airway manipulation	Airway secured but blocks reduce opioid need → partial respiratory protection	NA safest; GA + RA better than GA alone
Hemodynamics	Induction → myocardial depression and vasodilation; fluctuations common	Sympathectomy-induced hypotension; manageable with low-dose NA + vasopressors	GA effects + reduced surgical stress from blocks; still less stable than NA alone	Proactive vasopressor support is crucial
Cognition (delirium/POCD)	Higher risk due to sedatives, opioids, and polypharmacy	Lower risk if opioid/benzodiazepine sparing	Reduced opioids versus GA alone but GA-related delirium risk persists	NA superior; GA + RA intermediate
Analgesia	Systemic opioids required; short duration	Provides surgical anesthesia + extended analgesia (especially CSEA/CSA + PNBs)	Blocks (PENG/SIFI) provide strong postoperative analgesia; opioid-sparing	NA and GA + RA > GA alone
Mobility and ERAS	Delayed mobilization due to opioids and sedation	Facilitates early mobilization; aligns with ERAS	Blocks support early mobility despite GA use	NA most ERAS-friendly; GA + RA acceptable alternative
Technical aspects	Widely familiar; fast induction	Technically demanding in scoliosis/calcified spine	Combines the familiarity of GA with the analgesic benefits of blocks	GA + RA is the “middle ground” where NA is not feasible
Complications	Aspiration, pneumonia, and myocardial ischemia	Hypotension, rare PDPH, and high spinal with overdose	GA-related complications + rare block-related risks	NA has fewer complications overall
Mortality and outcomes	Similar 30-day mortality but ↑ morbidity and delayed functional recovery	Better functional outcomes and lower morbidity	Comparable to NA in pain outcomes; not superior in cognition or pulmonary	Functionally: NA > GA + RA > GA

Enhanced recovery and functional outcomes

Our patient’s mobilization by postoperative day 3 and discharge by day 7 highlight the efficacy of ERAS protocols [36]. Immobility is a key mortality predictor post-hip fracture, with early ambulation reducing pneumonia, deep vein thrombosis, and sarcopenia while promoting psychological well-being [68,69]. Our ERAS approach, integrating opioid-sparing analgesia, normothermia, and physiotherapy, proved effective even at 111 years [12,37,38]. The efficacy of our supercentenarian-specific ERAS pathway (Table 7), which facilitates early mobilization and discharge, is supported by Zhu et al. (2021), who demonstrated reduced complications and shorter hospital stays in elderly patients with intertrochanteric fractures using an ERAS protocol [70]. This case validates the concept that ERAS principles are not only feasible but indispensable in such age groups.

**Table 7:** Supercentenarian-specific enhanced recovery after surgery (ERAS) pathway. CSEA, combined spinal-epidural anesthesia; PENG, pericapsular nerve group; SIFI, supra-inguinal fascia iliaca

Phase	Intervention	Details	Rationale/Reference
Preoperative	Multidisciplinary assessment	Frailty scoring (e.g., Fried 3/5), optimize nutrition, and correct anemia	Tailored to supercentenarian frailty
Intraoperative	Neuraxial anesthesia (CSEA)	Ultra-low dose (0.6 mL 0.5% bupivacaine) and PENG/SIFI blocks	Minimizes hemodynamic instability and opioid use
	Hemodynamic support	Proactive norepinephrine (0.05 µg/kg/minute) and invasive monitoring	Prevents hypotension and ensures stability
	Normothermia maintenance	Forced-air warming (36.5°C-37°C)	Reduces complications
Postoperative	Multimodal analgesia	SIFI block (30 mL 0.2% ropivacaine) and no opioids	Preserves cognition and reduces delirium
	Early mobilization	Physiotherapy by day 1 and ambulation by day 3	Enhances recovery and reduces immobility risks
	Nutritional support	Early oral intake post-surgery	Addresses malnutrition and supports healing

Comparison with reported cases

Reports on centenarians and supercentenarians are scarce, but they consistently support RA for its safety and efficacy [10,26-28,48,50,52-55]. These reports highlight the role of ultra-low-dose RA in minimizing hemodynamic instability and delirium. Unlike these isolated cases, our comprehensive approach, integrating CSEA, PENG, and SIFI blocks, as well as proactive norepinephrine infusion and ERAS protocols, offers a reproducible template for supercentenarian trauma surgery. Our case, therefore, contributes to the novelty of the field, as it demonstrates how a structured, physiology-based strategy can yield successful outcomes in a supercentenarian trauma patient, providing a template for reproducibility rather than an isolated anecdote.

Broader ethical and clinical implications

A frequently asked question is whether surgery in centenarians/supercentenarians is justified. Critics argue that the risks outweigh the benefits, while proponents emphasize the quality of life and independence [71]. Literature increasingly supports the latter, showing that the surgical fixation of fractures in centenarians offers superior survival, pain control, and functional recovery compared to conservative management [9,47]. Respecting physiology over chronology is key, as it aligns interventions with patient goals [2]. Our patient’s recovery at 111 years demonstrates that protocol-driven anesthesia can restore mobility and the quality of life, challenging age-based biases [55,72]. Our patient’s recovery reinforces a critical principle: chronological age should never be the sole determinant of care.

Strengths and limitations

The major strength of this report lies in its novelty. To our knowledge, this is among the very few documented cases of hip fracture fixation in a supercentenarian above 110 years, managed with a structured, protocol-based perioperative pathway. Unlike most reports that describe isolated anesthetic techniques, this case integrates ultra-low intrathecal dosing, CSEA flexibility, proactive norepinephrine support, and multimodal regional blocks within an ERAS framework. This holistic, physiology-driven approach not only ensured intraoperative stability but also translated into early ambulation and discharge, providing a model of care that is reproducible across centers.

However, certain limitations must be acknowledged. Being a single case report, its findings cannot be generalized across all supercentenarians, who may present with greater frailty or significant comorbidities. Additionally, although outcomes were favorable, long-term follow-up beyond discharge was not available, which limited our ability to comment on sustained functional recovery. Finally, the absence of a comparator group (such as GA or conservative management) precludes definitive conclusions regarding the superiority of one technique over another. Nonetheless, the case highlights practical strategies, educational insights, and institutional protocols that may serve as a valuable reference point for anesthesiologists confronted with similar ultra-elderly patients worldwide.

## Conclusions

The successful anesthetic management of a 111-year-old supercentenarian redefines the boundaries of surgical feasibility in extreme age. A physiology-guided, protocol-driven approach, anchored in ultra-low intrathecal dosing, proactive norepinephrine-supported hemodynamic stabilization, and multimodal regional analgesia using PENG and SIFI blocks, ensured intraoperative stability, preserved cognition, and enabled early mobilization. Across literature from octogenarians to supercentenarians, regional anesthesia consistently demonstrates superiority over general anesthesia by reducing pulmonary complications and delirium and promoting functional recovery. This case reinforces that frailty and physiology, not chronology, must guide anesthetic decisions.

Three pillars underpin success in this demographic: titratable neuraxial safety (CSEA/CSA), opioid-free empowerment through peripheral blocks, and motion-driven recovery under ERAS principles. Ultimately, anesthesia for the ultra-elderly transcends survival; it is an art of restoration. This was not merely a procedure but a walk to remember, a testament that, with evidence-based precision and reverence for physiology, even supercentenarians can reclaim independence and dignity.
